# Quantification of Paeoniflorin by Fully Validated LC–MS/MS Method: Its Application to Pharmacokinetic Interaction between Paeoniflorin and Verapamil

**DOI:** 10.3390/molecules27238337

**Published:** 2022-11-29

**Authors:** Bin Bao, Yonglin Zhao, Huan Gong, Songshan Shi, Huijun Wang, Shunchun Wang

**Affiliations:** The MOE Key Laboratory for Standardization of Chinese Medicines and the SATCM Key Laboratory for New Resources and Quality Evaluation of Chinese Medicines, Institute of Chinese Materia Medica, Shanghai University of Traditional Chinese Medicine, 1200 Cailun Road, Shanghai 201203, China

**Keywords:** paeoniflorin, verapamil, pharmacokinetic, LC–MS/MS

## Abstract

A rapid, sensitive, and specific LC-MS/MS method was developed and fully validated for the detection of paeoniflorin only in rat plasma, and applied to pharmacokinetic studies, including intravenous, multi-dose oral and combined administrations with verapamil. In this study, tolbutamide was used as the internal standard, and the protein precipitation extraction method, using acetonitrile as the extraction agent, was used for the sample preparation. Subsequently, the supernatant samples were analyzed on a Phenomenex Gemini^®^ NX-C18 column with a flow rate of 1.0 mL/min in a gradient elution procedure. In the extracted rat plasma, the method exhibited high sensitivity (LLOQ of 1.0 ng/mL) upon selecting ammonium adduct ions ([M+NH_4_]^+^) as the precursor ions and good linearity over the concentration range of 1.0–2000 ng/mL, with correlation coefficients >0.99. The intra- and inter-batch accuracy RE% values were within ±8.2%, and the precision RSD% values were ≤8.1% and ≤10.0%, respectively. The results show that the method can be successfully applied to quantitate paeoniflorin in biological samples. Additionally, paeoniflorin is subsequently confirmed to be the substrate of the P-gp transporter in vivo and in vitro for the first time, which would be necessary and beneficial to investigate the clinical safety and efficacy of PF with other drugs in the treatment of rheumatoid arthritis.

## 1. Introduction

At present, rheumatoid arthritis (RA) is a chronic, systemic inflammatory disease that affects 0.2–0.4% of Chinese adults, an estimated 5 million people [1]. It is more commonly present in women and can occur at any age, with the highest incidence occurring between the ages of 40 and 60 years. Additionally, RA is characterized by joint pain and swelling, which can severely impair an individual’s physical function and quality of life. Compared with the general population, people with RA are at greater risk for serious infections, respiratory disease, osteoporosis, cardiovascular disease, cancer, and death [2]. RA is affected by multiple factors and is also primarily characterized by synovitis. Retrospective studies conducted on RA pathogenesis showed genetic and environmental factors as strong inducers of RA. Based on RA pathogeneses, multifarious anti-RA drugs, particularly non-steroidal anti-inflammatory drugs (NSAIDs), are the most commonly used in the treatment of RA and inflammatory-related diseases [3,4]. However, NSAID users showed presented hepatotoxicity, cardiovascular risks, as well as renal events in patients, especially in old patients with related primary diseases [5,6,7]. Moreover, it is expensive and unavailable to some poor countries and regions. In the last two decades, natural products are receiving increasing attention as they have become one of the best choices for chemists searching for novel agents or active templates for use in the field [8]. In addition, natural products (e.g., flavonoids, phenolics, triterpenoids, and alkaloids) are more accessible, relatively safer to use, and cost-effective. It has been proven that various herbal medicines and their active components present anti-RA effects, mainly due to their anti-inflammatory, antioxidant, and apoptosis mechanisms [9,10]. Curcumin, a natural product, has similar efficacy properties to diclofenac but demonstrates better tolerance among patients with knee osteoarthritis [11]. In this case, natural products can be an alternative treatment option for patients with inflammatory diseases who are intolerant to the side effects of NSAIDs.

**Figure 1:**
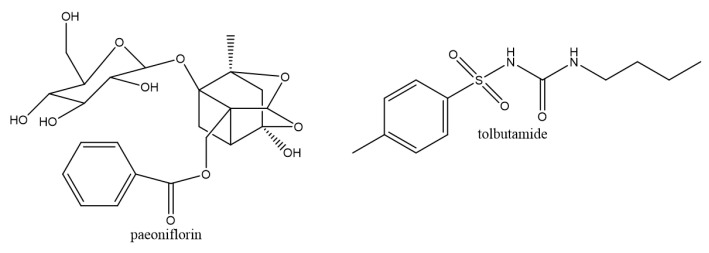
Chemical structures of paeoniflorin and tolbutamide (IS).

The total glucosides of paeony (TGP), mainly consisting of paeoniflorin (PF, Figure 1), albiflorin, paeonin, oxy-paeoniflorin, and benzoyl-paeoniflorin, was reported to have remarkable benefits for RA [12,13]. Additionally, total glucosides of paeony capsules (TGPCs) were approved by the China Food and Drug Administration in 1998 as an anti-inflammatory and immunomodulatory drug for rheumatoid arthritis (RA) patients [14]. Many researchers have previously demonstrated that PF exerts anti-inflammatory and autoimmune regulatory effects through multiple targets [12,15]. In comparison to the existing treatments in the field, TGP and PF present some superior features with few adverse reactions and multiple mechanisms, including anti-inflammatory activity, beneficial effects on monocytes and macrophages, and regulating signal pathways [15,16]. Therefore, TGP and PF have a broad application prospect in the treatment of RA. However, numerous natural products are in classes III/IV of the biopharmaceutics classification system (BCS), presenting low-permeability properties. For classic BCS, paeoniflorin is in class III; rutin, hyperoside, and salvianolic acid B are in classes III/IV and nuciferine is in classes III/IV, based on the everted gut sac assay [17]. In addition, PF is a water-soluble monoterpene glycoside, resulting in its poor permeability property due to its molecule containing a small glycoside and numerous polar groups. Consequently, it is necessary to do further study for the causes of the poor absorption properties of natural products.

P-gp, an ATP-powered efflux pump, can deliver hundreds of structurally unrelated hydrophobic amphoteric compounds, including therapeutic drugs, peptides, and lipids [18]. Numerous commonly used drugs, including vinblastine, taxanes, tyrosine kinase inhibitors, and some PARP inhibitors, were p-glycoprotein substrates. In the gut, P-gp, expressed on the apical surface of epithelial cells of the large and small intestines in high levels, was responsible for venting drugs into the lumen, thereby decreasing drug absorption and oral bioavailability. Some researchers revealed that PF has low bioavailability properties due to its poor intestinal permeability [19]. Additionally, the efflux ratio (P_app AB_/P_app BA_) of PF was higher than two in the Caco-2 cell-permeability experiment, indicating that some transporters might be involved in its disposal process [20,21]. Verapamil, an anti-hypertensive calcium channel blocker, was observed to be a P-gp inhibitor [22]. For the purpose of illustration, the oral AUC and bioavailability of the P-gp substrate (e.g., Aliskiren, Betrixaban, and Celiprolol) tended to be relatively higher in P-gp-lacking than wild-type mice, along with a higher C_max_ value [23]. The effect of verapamil on PF in animals has not yet been reported in the literature; therefore, it is necessary to confirm whether PF is the substrate of P-gp and influences the bioavailability of PF.

In recent years, mass spectrometry, particularly liquid chromatography tandem mass spectrometry (LC–MS/MS), has emerged as a powerful tool for the detection and characterization of PF as a result of its high sensitivity and specificity characteristics [24,25]. Our validated LC–MS/MS method has a wider linear range and greater sensitivity, along with a better ability to store samples, its economical column, and instrument-friendly flow rate, which can be transferred more successfully to disparate laboratories and instruments.

It is well-known that nonclinical pharmacokinetic data obtained from animals are of considerable importance and directive significance for the clinical development of drugs. The purpose of the present study was to determine whether PF is the substrate of P-gp in animals and confirm that P-gp mediates the absorption process of paeoniflorin, and finally explain the poor absorption rate of paeoniflorin from this perspective. Hence, a rapid, sensitive, and specific LC-MS/MS method was successfully established and validated in the analysis of plasma samples obtained from the pharmacokinetic studies of PF in SD rats. The present research provides the foundations for the transformation of PF into clinical drugs.

## 2. Results and Discussion

### 2.1. Method Development

**Figure 2:**
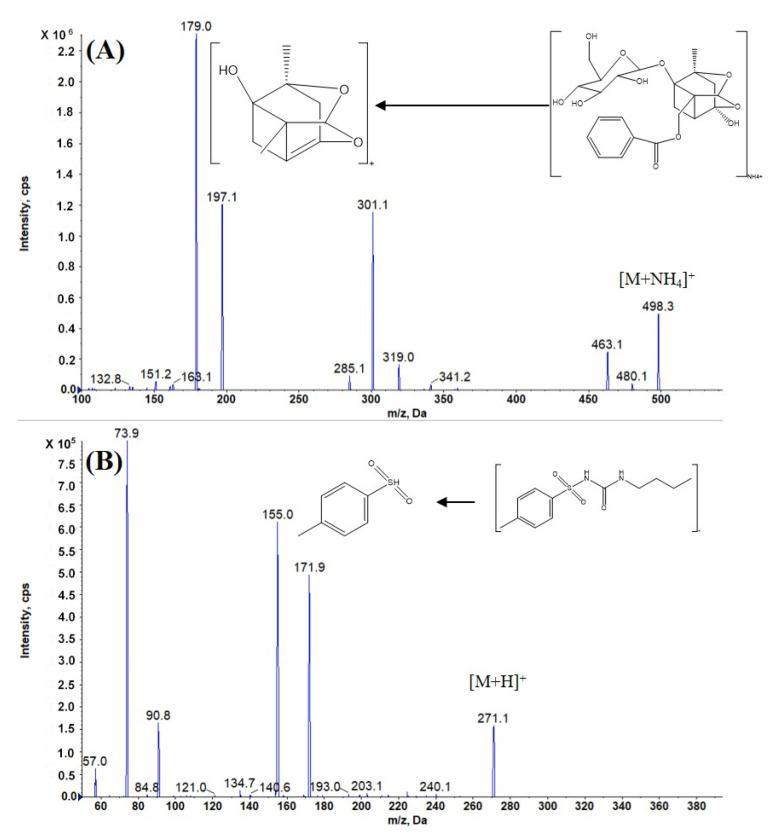
Positive product ion mass spectra of PF (**A**) and IS (**B**) and their proposed fragmentation patterns.

The method operated on AB Sciex Qtrap 5500; tolbutamide, with its stable signal strength and broad application, was selected as the internal standard. For the optimization of the sensitivity and specificity characteristics, the multiple-reaction monitoring (MRM) mode was selected for the data collection process. According to the Q1 full-scan mass spectra, the protonated molecule ion [M+H]^+^ was invisible for IS and was also in low abundance for PF under the positive ESI mode. Subsequently, the precursor [M+NH_4_]^+^ ions were fragile so that they could be fragmented to generate abundant product ions with a high yield for PF. In addition, the Q1 full-scan mass spectra in the negative mode were also conducted on PF and the precursor [M−H]–ions, with an intensity approximately 200 times lower than in the positive-ion mode. On the basis of this result, to generate a high yield of stable and dominating product ions from [M+NH_4_]^+^, mild-collision-induced dissociation (CID) conditions, such as low collision energy (CE) and medium CAD, were applied. The final MRM transitions used for the quantification process were *m*/*z* 498.1 → 179.1, 271.1 → 154.9 for PF and IS (Figure 2), respectively. Other mass spectrum parameters were also optimized and are presented in Table 1.

The liquid chromatography conditions of the mobile phase, salt type, flow rate, and oven temperature were investigated. A Phenomenex Gemini^®^ NX-C18 column (4.6 × 100 mm, 3.0 μm) was applied for the sample analysis process due to its good durability property, wide applicability, and low residue of analytes. Following the optimization of the peak, resolution, and stable ammonium signal output, aqueous (0.1% formic acid and 5 mM of ammonium acetate in distilled water) and organic (acetonitrile) phases were used as the mobile phase at a flow rate of 1.0 mL/min, with gradient elution.

Protein precipitation, one of the frequently used extraction procedures in the field, was elected for the plasma-sample preparation, in terms of its cheap cost and efficiency [26]. Acetonitrile was selected to perform the extraction of compounds, due to its better response when compared to methanol. Finally, a rapid and economic method was applied to the following method validation and in vivo experiments.

### 2.2. Method Validation

The LC-MS/MS method was validated to perform the bioanalysis of PF in the SD rat plasma samples. There were seven analysis batches for the plasma, including items of selectivity, linearity, precision and accuracy, recovery, matrix effect, stability, and dilution integrity.

#### 2.2.1. Selectivity and Interference

Individual blank plasma samples, blank plasma-added IS and ULOQ samples without IS, and corresponding LLOQ samples were prepared according to the sample-preparation procedure described above and screened for any interference. As can be observed in Table 2, the peak area of endogenous interference was not greater than 20% and 5% of that of LLOQ and IS, respectively.

**Figure 3:**
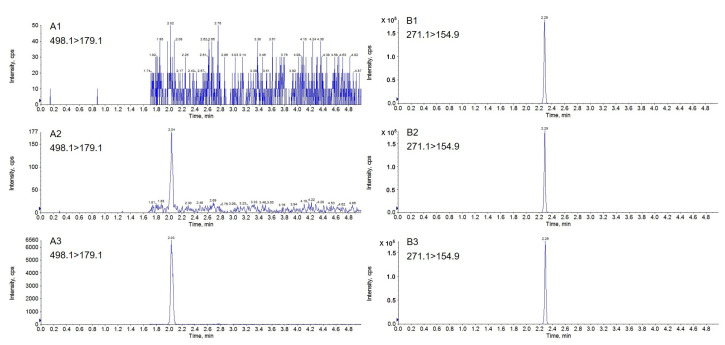
Typical chromatograms of PF (**A1**–**A3**) and IS (**B1**–**B3**) for (1) double blank plasma sample, (2) extracted plasma sample at lower limits of quantification (LLOQ) and (3) extracted plasma at 0.25 h after mono-administration of PF.

The analyte and IS, meeting the acceptance criteria of selectivity, also presented no interference with each other. Typical chromatograms of PF (A) and IS (B) samples in the plasma are presented in Figure 3.

#### 2.2.2. Standard Curve and Linearity

For the plasma, a calibration curve was constructed from 1 to 2000 ng/mL for PF by the best linear fitting of peak area ratios (PF to IS) vs. concentrations with a weighting factor of 1/x^2^. As presented in Table 3, the RE% values between the back-calculated concentrations of calibration samples and their nominalized concentrations were within ±9.2%. The present LC-MS/MS method exhibited good linearity (r > 0.9972) using the equation of linear regression (y = 0.000868x + 4.96 × 10^−5^. By gradually diluting the sample of a known concentration, a concentration point with a signal-to-noise ratio (S/N) of 10 was obtained. By taking the corresponding variation in the instrument and the different responses of the diverse matrix into account, we selected a limit of quantitation of approximately five times as the sensitivity factor (1 ng/mL, RE% and RSD% values were within 5.0%, 7.7%, respectively, S/N = 45.5), along with the limit of detection (LOD, 0.1 ng/mL), to be calculated.

#### 2.2.3. Precision and Accuracy

The precision and accuracy values of the LC-MS/MS method were estimated over four QC levels. As presented in Table 4, the plasma-determination results for the precision and accuracy values showed that the intra- or inter-batch precision RE% and accuracy RSD% values were within ±5.4%, and 10%, respectively.

#### 2.2.4. Carryover Effect

The peak area of a double-blank sample right immediately following the injection of ULOQ and high-QC samples was not higher than 20% of LLOQ and approximately 0.5% of IS, respectively, meeting the acceptance criteria of the carryover effect.

#### 2.2.5. Matrix Effect and Recovery

As presented in Table 5, the plasma-determination results show that the mean IS-normalized matrix factor at low-, middle-, and high-QC levels were 1.04, 1.11, and 0.99, respectively, and the RSD% values at each concentration were 8.3%, 4.9%, and 4.2%, which met the acceptance criteria. Additionally, the results show that the matrix effect did not affect the quantitative results obtained for the biological samples.

As presented in Table 5, the plasma-determination results show that the mean IS-normalized recovery rates at low-, middle-, and high-QC levels were 97.0%, 93.0%, and 98.0%, respectively, and the RSD% values at three concentrations were not higher than 5.3%.

#### 2.2.6. Stability

Stability in the plasma was tested by comparing the two-level QC samples (LQC and HQC) in sextuplicate injections to theoretical concentrations. As displayed in Table 6, the RE% values determined in the plasma at each concentration level were within ±10.8%, and the RSD% values were not higher than 6.0%, respectively, indicating that the PF was stable in the SD rat plasma following 16 h lying on bench-top at an ambient temperature, post-precipitation storage in an auto-sampler for 72 h at an ambient temperature, 4 freeze cycles at −20 °C then thawing at room temperature, and 90-day storage in a −20 °C freezer.

#### 2.2.7. Dilution Integrity

As presented in Table 7, the upper limit of the LC-MS/MS method could be appropriately extended to 15,000 ng/mL of PF in SD rat plasma samples, as having an RSD of 2.8% and n RE of 0.6% for the determination of PF in SD rat plasma samples following a 10-fold dilution of the DQC samples.

### 2.3. Effect of Verapamil on the Transport of Paeoniflorin

**Figure 4:**
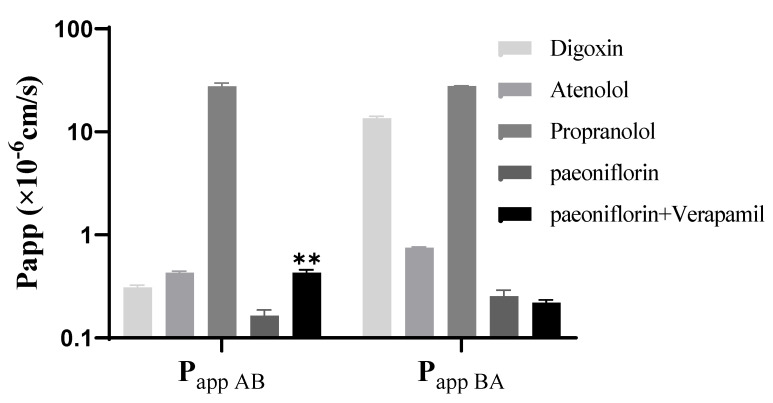
The transport of PF from apical (AP) to basolateral (BL) side and the opposite direction with the treatment of verapamil. Each symbol represents the mean ± SD of two determination. Three quality control samples of atenolol, propranolol and digoxin represented low permeability, high permeability and potential P-gp substrates, respectively.

The P_app AB_ and P_app BA_ values of PF in the absence of verapamil were 0.17 × 10^−6^ cm/s and 0.26 × 10^−6^ cm/s, respectively, and the efflux ratio was calculated as 1.52. In the presence of verapamil, a specific inhibitor of P-gp activity, the value of P_app AB_ of PF increased to 0.43 × 10^−6^ cm/s, while the value of P_app BA_ presented a significant change with an efflux ratio of 0.52. Thus, verapamil significantly enhanced the influx ratio of PF from 0.17 to 0.43, suggesting that PF may be a substrate for the P-gp efflux transporter (*p* < 0.05, Figure 4).

### 2.4. Pharmacokinetic Study

**Figure 5:**
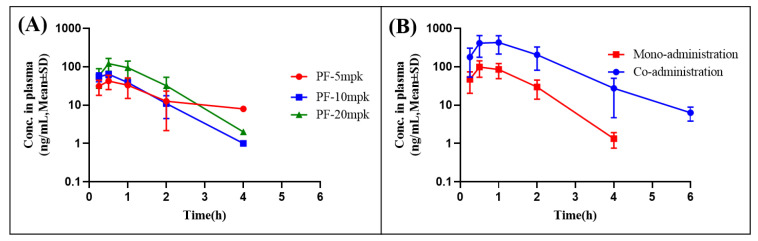
Mean± SD plasma concentration-time profiles of PF after administration (5, 10, 20 mg/kg) in SD Rat (**A**); Mean± SD plasma concentration-time profiles of PF after mono- and co-administration in SD Rat (**B**).

The fully validated method was successfully applied for determining a pharmacokinetic study on male Sprague Dawley (SD) rats following gavage and intravenous administration, which was subsequently adapted to the mono-administration of PF and its co-administration with verapamil. The mean plasma concentrations vs. time profiles of PF in male SD rats are presented in Figure 5, and the corresponding pharmacokinetic parameters are depicted in Table 8.

Following the single-dose administration of PF using oral and tail-vein injections, the pharmacokinetic parameters of the AUC_0–t_ for oral administration were 64.7 ± 44.0, 82.3 ± 18.4, and 148.6 ± 64.8 ng·mL^−1^ separately, and the AUC_0–t_ for the intravenous administration was 2401.0 ± 201.9 ng·h·mL^−1^. Subsequently, these data were integrated and finally calculated the absolute oral bioavailability of PF, ranging from 0.6% to 1.3%, which was attributed to the rapid absorption rate (T_max_: 0.5 ± 0.0 h ~ 0.7 ± 0.3 h), quick metabolism rate (T_1/2_: 0.5 ± 0.1 ~ 0.8 ± 0.4 h, CL/F: 98.2 ± 77.9 ~ 145 ± 54.9 L·h^−1^·kg^−1^), and low tissue distribution (V_z_: 0.2 ± 0.0 L·kg^−1^), indicating that the oral bioavailability of PF was extremely low. In addition, according to Table 8, following multidose oral administration of PF, the pharmacokinetic parameters of C_max_ were 42.3 ± 17.5, 63.4 ± 11.4, and 100.2 ± 42.5 ng·h·mL^−1^, respectively. It can be observed from these data that the peak plasma concentration did not increase in proportion with the elevated dose, which showed that PF presented the phenomenon of absorption saturation at high doses. PF is a monoterpenoid glycoside compound with high solubility and low permeability characteristics [17]. Previous research showed that the transport rate of PF through the Caco-2 monolayer cell was quite low and the efflux rate constant was higher than 2, indicating that PF may be the substrate of the intestinal efflux transporter [21]. Taking the above data into consideration, the co-administration of a high oral dose and verapamil, a P-gp inhibitor, was selected to verify PF as the substrate of the P-gp transporter in vivo, meanwhile proving that the efflux ratio decreased by verapamil was one of the reasons for the poor absorption of PF.

According to the statistical results of the pharmacokinetic parameters of the co-administration group following the oral administration of PF combined with verapamil presented in Table 8, we compared the pharmacokinetic parameters of PF with the mono-administration group. The values of C_max_, AUC_0–t_ and AUC_0–∞_ in the co-administration group dramatically increased 4.6 times (*p* < 0.01), showing that the absorption value of paeoniflorin also increased. Additionally, the values of AUC_0–t_ and AUC_0–∞_ in the co-administration group also increased 5.9 and 5.7 times (*p* < 0.05), respectively, showing that the verapamil-mediated inhibition of P-gp transporters was a relatively long-term effect, along with greater oral bioavailability values. The statistical analysis presented no significant difference in the values of T_max_, T_1/2,_ and MRT_0–∞_. The above-mentioned data show that, by the oral co-administration of PF and verapamil, the latter affects the pharmacokinetic parameters of PF by increasing the absorption degree, the bioavailability, and reducing the rate of drug elimination. Previous studies have reported that P-gp was involved in mediating the absorption of PF [27,28]. Following the co-administration of the drugs, verapamil inhibited the expression of intestinal P-gp and weakened its efflux effect on PF, thus increasing the absorption and bioavailability of PF, along with indirectly mitigating the elimination rate of PF.

## 3. Materials and Methods

### 3.1. Chemicals and Reagents

PF (Lot: ST007001, purity: 99.4%) was purchased from Nature Standard (Shanghai, China). Tolbutamide (IS, Lot: 100369-201307, purity: ≥ 98.0%) was purchased from Yuanye Biotechnology (Shanghai, China). Verapamil Hydrochloride (Lot: LGVBJ-ET, purity: 98.0%) was subscribed from TCI (Shanghai, China). The structure of PF and tolbutamide are shown in Figure 1. Acetonitrile, methanol and dimethyl sulfoxide, up to HPLC grade, were purchased from Merck (KGaA, Darmstadt, Germany). Deionized water was collected from a Milli-Q ultra-pure grade water system (Millipore Corp., Massachusetts, MA, USA). All the other chemicals were of analytical or HPLC grade.

### 3.2. Animals

Male Sprague–Dawley rats with a body weight of 160 ± 20 g were purchased from Shanghai Xipu-Bikai Experimental Animal Co., Ltd., (Shanghai, China). All animals were fed adaptively in a constant temperature (23 ± 2 °C) and humidity (55 ± 10%) environment for one week before the experiment. Animal studies were carried out in accordance with the Guide for the Care and Use of Laboratory Animals as adopted and promulgated by the National Health Ministry of China.

### 3.3. LC-MS/MS Conditions

HPLC was conducted on a Shimadzu LC-30AD (Kyoto, Japan) series instrument. A Phenomenex Gemini^®^ NX-C18 column (4.6 × 100 mm, 3.0 μm) was used for the separation. Two solvents were used for gradient elutions: (A) 5 mM ammonium acetate and 0.1% formic acid (FA) in water, and (B) acetonitrile. The linear gradient used was as follows: 0 min, 10% B; 0.3 min, 10% B; 1.0 min, 98% B; 4.0 min, 98% B; 4.1 min, 10% B and 5.0 min, 10% B. The flow rate was set at 1.0 mL/min, and the column temperature was maintained at 40 °C. The injection volume of the automatic sampler was 20 μL.

Tandem mass spectrometry analysis was performed on a QTRAP 5500 hybrid triple quadrupole mass spectrometer (AB Sciex, Framingham, MA, USA), coupled at an electrospray ion source (ESI), with Analyst software 1.7.0 workstation used for data acquisition, and qualitative and quantitative analyses. Nitrogen was used as curtain (CUR) gas and collision activation dissociation (CAD) gas. MS spectra were acquired in the positive MRM mode. MS conditions were optimized as follows: Ion Spray Voltage +5000 V; source temperature 550 °C and dwell time 100 ms. CUR, GS1, and GS2 were set at 40, 55, and 55 psi, respectively. The compound-dependent instrumental parameters, including precursor ion, product ion, decluttering potential (DP), entrance potential (EP), collision energy (CE), and collision cell exit potential (CXP), were optimized and are summarized in Table 1.

### 3.4. Sample Preparation

All plasma samples were allowed to thaw at ambient temperature. An aliquot of 40 μL of each plasma sample was dispensed into a 2.0 mL 96-deep well plate, followed by adding 200 μL of the IS working solution (50 ng/mL) or acetonitrile (preparing the double-blank sample). The mixture was vortexed at 1000 rpm for 5 min and then centrifuged at 6000 rpm for 20 min at 4 °C. An aliquot of 180 μL of each supernatant was transferred to a clean tube, along with a 20 μL sample being injected into the LC-MS/MS for analysis.

### 3.5. Method Validation

The following parameters were investigated based on the guidelines for the bioanalytical method validation set by the US Food and Drug Administration: selectivity, linearity and sensitivity, accuracy and precision, extraction recovery and matrix effect, stability, dilution factor, and carryover.

#### 3.5.1. Stock and Working Solutions, Calibration Standards, and Quality Control Samples

A PF stock solution of 1 mg/mL was prepared by dissolving an accurately weighted amount of PF in an appropriate volume of DMSO. The stock solution was prepared twice and named the PF standard stock solution (PSS) and PF quality control stock solution (PQS), respectively. The IS stock solution of 1 mg/mL was prepared by dissolving an accurately weighted amount of Tolbutamide in an appropriate volume of DMSO. All the stock solutions were stored in a −20 °C freezer prior to use.

The IS working solution was diluted by the IS stock solution with acetonitrile to yield a final concentration of 50 ng/mL and was stored at a −20 °C freezer prior to use.

The PSS was removed and diluted with methanol: H_2_O (1:1, *v*/*v*) to prepare the standard curve working solutions of 0.02, 0.04, 0.1, 1, 4, 20, 32, and 40 μg/mL. The calibration curve samples of PF were prepared by spiking blank plasma with each working solution to yield concentrations of 1, 2, 5, 50, 200, 1000, 1600, and 2000 ng/mL.

The PQS was removed and diluted with methanol: H_2_O (1:1, *v*/*v*) to prepare the quality control working solutions of 0.02, 0.06, 3.0, 30, and 300 μg/mL. Quality control samples of PF were prepared by spiking blank plasma with each working solution to yield concentrations of five different concentrations, a lower limit of quantification (LLOQ, 1 ng/mL), low-level quality control (LQC, 3 ng/mL), medium-level quality control (MQC, 150 ng/mL), high-level quality control (HQC, 1500 ng/mL), and dilution quality control (DQC, 15,000 ng/mL).

#### 3.5.2. Specificity

The specificity was measured by analyzing blank plasma samples collected from six different donors, blank plasma samples spiked with PF and IS, and plasma samples obtained following oral administration, to exclude any endogenous interference or interference close to the expected retention times of PF and IS.

#### 3.5.3. Linearity and Sensitivity

The calibration curve was constructed by plotting the analyte-to-IS-peak-area ratios against analyte concentration. The linearity was investigated by establishing a weighted (1/x^2^) least squares regression. The calibration curve required a correlation coefficient (r) of ≥ 0.995. LLOQ was defined as the lowest concentration of the calibration curve with S/N > 5.

#### 3.5.4. Carryover Effect

The carryover effect of PF was evaluated by analyzing blank plasma samples after determining the highest concentration point of the calibration curve and was repeated three more times. Interference should be < 20% of LLOQ for PF and 5% for IS. Moreover, the samples exceeding the standard values were less than one third of the total samples.

#### 3.5.5. Accuracy and Precision

The intra- and inter-day accuracy and precision values were assayed by analyzing six replicate QC samples at LLOQ, low, medium, and high concentrations on the same day and three consecutive days, respectively. The relative standard deviation (RSD, %) for the precision should be < 15%, and the acceptable relative error (RE, %) for the accuracy was required to be within ±15% of the nominal values.

#### 3.5.6. Matrix Effects and Extraction Recovery

The Matrix effect was measured by comparing the peak areas of post-extraction blank plasma samples spiked with PF with those of standard solutions at the same concentrations. The blank plasma samples were collected from six different donors (*n* = 6). The values of the matrix effect should be within the range of 85~115%. The extraction recovery and matrix effect were evaluated by QC samples at three concentration levels (3, 150, and 1500 ng/mL, respectively). The extraction recovery of PF was evaluated by comparing the peak areas of PF from pre-extracted QC samples with those of samples reconstituted with blank extracted plasma (post-extraction) at the same concentrations. The extraction recovery and matrix effect of IS were determined in the same procedure.

#### 3.5.7. Stability

The stability of PF in rat plasma was investigated by analyzing QC samples at three concentrations. Long-term stability was tested at −20 °C for at least 30 days; short-term stability was tested at room temperature for at least 6 h; the post-preparative stability test was conducted at 10 °C for 72 h in an autosampler and freeze–thaw stability was determined after 3 freeze (−20 °C) and thaw cycles. The determined concentrations should be within ±15% of the nominal concentrations.

#### 3.5.8. Dilution Factor

The effect of dilution was evaluated by diluting six replicates of QC samples at a concentration of 15 μg/mL with blank rat plasma to yield the final concentration of 1.5 μg/mL. The diluted samples were processed and determined as above. The accuracy should be within ±15% of the nominal concentration with RSD < 15%.

### 3.6. Cell Culture and Caco−2 Transwell Model

The Caco−2 cells were purchased from ATCC (Manassas, VA, USA) and cultured in a DMEM high-glucose medium with 15% FBS, 1% NEAA, and 100 U/mL penicillin and streptomycin. A total of 1.25 × 10^5^ cells/cm^2^ were seeded into polyethylene membranes (PET) in 96-well Falcon insert systems for 21–28 days to obtain a confluent cell monolayer.

The test compounds were diluted with the transport buffer (HBSS with BSA) obtained from a 10 mM stock solution to a concentration of 10 µM and applied to the apical or basolateral sides of the cell monolayer. The permeation of the test compounds from A to B directions or B to A directions was determined in duplicate over a 120-min incubation period at 37 °C and 5% CO_2_ with a relative humidity of 95%. In addition, the efflux ratio of each compound was also determined. Test and reference compounds were quantified by LC-MS/MS analysis based on the peak area ratio of analyte/IS.

The apparent permeability coefficient, Papp (cm/s), was calculated using the following equation:

P_app_ = (dCr/dt) × Vr/(A × C0)

where dCr/dt is the cumulative concentration of the compound in the receiver chamber as a function of time (S); Vr is the solution volume in the receiver chamber (0.1 mL on the apical side and 0.25 mL on the basolateral side); A is the surface area for the transport, i.e., 0.0804 cm^2^ for the area of the monolayer and C0 is the initial concentration in the donor chamber.

The efflux ratio was calculated using the following equation:

Efflux Ratio = P_app BA_/P_app AB_

where P_app BA_ is the apparent permeability coefficient obtained from the BL to AP sides (cm/s), and P_app AB_ is the apparent permeability coefficient obtained from the AP to BL sides (cm/s).

The percent recovery was calculated using the following equation:

% Recovery = 100 × [(Vr × Cr) + (Vd × Cd)]/(Vd × C0)

% Total recovery =100 × [(Vr × Cr) + (Vd × Cd) + (Vc × Cc)]/(Vd × C0)

where Vd is the volume in the donor chambers (0.1 mL on the apical side and 0.25 mL on the basolateral side); Cd and Cr are the final concentrations of the transport compound in the donor and receiver chambers, respectively. Cc is the compound concentration in the cell lysate solution. Vc is the volume of the insert well.

### 3.7. Pharmacokinetic Study

The animal experiment was approved by the Experimental Animal Ethics Committee of Shanghai University of Traditional Chinese Medicine (the Ethics Number: PZSHUTCM201016015). After 7 days of freely feeding the animals to acclimation, the rats were randomly divided into five groups, including the following: PF at an intravenous dose of 2 mg/kg and intragastric administrations of 5 mg/kg, 10 mg/kg, and 20 mg/kg; PF combined with verapamil at doses of 20 mg/kg and 50 mg/kg, respectively. All groups of PF or PF combined with verapamil were dissolved in saline to be completely dissolved or suspended. Blood samples were collected into EDTA−2K anti-coagulant tubes at projected times (0.083, 0.25, 0.5, 1, 2, 4, and 6 h post-dose) and then immediately centrifuged at 5500 rpm at 4 °C for 10 min to obtain the supernatant. The plasma samples were stored at −20 °C prior to analysis.

### 3.8. Data Analysis

The plasma drug concentration of the PF was quantitated, according to the calibration curve, by the ratio of the analyte-to-IS peak areas obtained for each sample. Pharmacokinetic parameters, such as elimination half-life (T_1/2_), the area under the concentration–time curve from time zero to infinity (AUC_0–∞_), total body clearance (CL), and volume of distribution (V_z_), were analyzed by the non-compartment model using WinNonlin software (Version 8.2). Subsequently, the main pharmacokinetic parameters were analyzed by an independent sample *t*-test. The oral absolute bioavailability was calculated as prescribed by the following equation: F (%) = (AUC_p_._o_. × Dose_i_._v_.)/(AUC_i_._v_. × Dose_p_._o_.) × 100%.

## 4. Conclusions

A rapid, sensitive, and fully validated LC–MS/MS method was established for the determination of PF. Subsequently, the method was successfully applied to the present pharmacokinetic study, including the permeability of Caco-2 cells in vitro, oral multidose, and intravenous administration, to calculate the bioavailability and effects of verapamil on the pharmacokinetic properties of PF. The results of the pharmacokinetic parameters show that PF exerts absorption saturation at a high dose and presents poor absorption properties, which were some of the reasons for the low bioavailability of PF. The co-administration of PF and verapamil demonstrated that P-gp mediated the intestinal absorption of PF, which was confirmed to be the substrate of the P-gp transporter in vivo and in vitro for the first time. Clinically, close attention should be paid to PF in combination with other Chinese herbal medicines, along with the clinical adverse reactions and side effects that occur. In addition, further in vitro research should investigate the pharmacokinetic properties of PF and be reported in the literature in the future.

## Figures and Tables

**Table 1:** Mass spectrometric parameters for paeoniflorin and tolbutamide (IS).

Compound	Q1	Q3	Dwell Time (msec)	RT (min)	DP (V)	CE (V)	EP (eV)	CXP (V)
Paeoniflorin	498.1	179.1	100	2.3	80	26	10	14
Tolbutamide	271.1	154.9	100	2.1	80	24	10	14
Scan Type				MRM				
Ionization Model				positive				
Ion Source				ESI				
CAD				Medium				
Curtain Gas				40 psi				
GS1				55 psi				
GS2				55 psi				
Ion Spray Voltage				5000 V				

**Table 2:** Selectivity and Interference of PF and tolbutamide (IS).

Sample	Analyte Peak Area	IS Peak Area	Analyte Peak	IS Peak Area (%)
			Area (%)	
Selectivity Blank-1	156	9730	7.16	0.42
Selectivity Blank-2	315	11,400	14.46	0.49
Selectivity Blank-3	348	9580	15.98	0.41
Selectivity Blank-4	267	10,100	12.26	0.43
Selectivity Blank-5	277	10,000	12.72	0.43
Selectivity Blank-6	153	9410	7.02	0.40
Mean LLOQ	2178	2,328,333	11.60	0.43
Only IS-1	0.000124	2,490,000	0.00	/
Only IS-2	0.0000245	2,480,000	0.00	/
Only IS-3	0.000114	2,490,000	0.00	/
Only ULOQ-1	4,770,000	14,000	/	0.56
Only ULOQ-2	4,730,000	11,700	/	0.47
Only ULOQ-3	4,760,000	11,700	/	0.47

**Table 3:** Calibration curves of the LC-MS/MS method for PF in SD rat plasma.

Nominal Conc. (ng/mL)	Intra-Batch (*n* = 3)		
	Measured Conc. (ng/mL)	Precision (RSD%)	Accuracy (RE%)
1.0	1.02 ± 0.01	1.1	2.3
2.0	1.94 ± 0.02	1.0	−3.0
5.0	4.81 ± 0.36	7.5	−3.9
50	45.9 ± 2.1	4.5	−8.3
200	195 ± 6.4	3.3	−2.3
1000	980 ± 25.0	2.5	−2.0
1600	1723 ± 51.3	3.0	7.7
2000	2183 ± 20.8	1.0	9.2

**Table 4:** Precision and Accuracy of the LC-MS/MS method for quantification of PF in SD rat plasma.

Nominal Conc. (ng/mL)	Intra-Batch (*n* = 6)			Inter-Batch (*n* = 3 × 6)		
	Measured Conc. (ng/mL)	Precision (RSD%)	Accuracy (RE%)	Measured Conc. (ng/mL)	Precision (RSD%)	Accuracy (RE%)
1.0	1.1 ± 0.09	8.1	8.2	1.0 ± 0.1	7.7	5.0
3.0	3.2 ± 0.22	6.8	6.7	3.0 ± 0.3	10.0	0.6
150	140.8 ± 8.5	6.0	−6.1	141.9 ± 10.4	7.3	−5.4
1500	1570 ± 78.2	5.0	4.7	1551.8 ± 89.0	5.7	3.5

**Table 5:** Recovery and Matrix Effect of PF in SD rat plasma (*n* = 6).

Nominal Conc. (ng/mL)	Extraction Recovery (%)		Matrix Effect (%)	
	Mean ± SD	RSD%	Mean ± SD	RSD%
3.0	97.0 ± 2.1	2.2	104.0 ± 8.6	8.3
150	93.0 ± 4.9	5.3	111.3± 5.5	4.9
1500	98.0 ± 3.9	4.0	99.3 ± 4.2	4.2

**Table 6:** Stability of PF in SD rat plasma under diverse storage conditions (*n* = 6).

Item	Storage Conditions	Nominal Conc. (ng/mL)	Measured Conc. (ng/mL)	Precision (RSD%)	Accuracy (RE%)
Bench-top Stability	Room temperature for 16 h	3.0	3.01 ± 0.21	7.1	0.3
		1500	1538 ± 52.0	3.0	2.6
Autosampler Stability	10 °C for 72 h	3.0	2.91 ± 0.16	5.4	−3.1
		1500	1535 ± 54.7	3.6	2.3
Freeze-thaw Stability	4 Cycles at −20 °C	3.0	3.14 ± 0.17	5.5	4.6
		1500	1662 ± 44.5	2.7	10.8
Long-term Stability	−20 °C for 90 days	3.0	2.86 ± 0.20	6.0	−4.8
		1500	1500 ± 37.4	2.5	0.0

**Table 7:** Dilution integrity for quantification of PF in SD rat plasma (*n* = 6).

Dilution Factor	Nominal Conc. (ng/mL)	Measured Conc. (ng/mL)	Precision (RSD%)	Accuracy (RE%)
10	15,000	14,917 ± 417	2.8	−0.6

**Table 8:** Pharmacokinetic parameters of PF in rat plasma after intravenous administration (2 mg/kg), oral mono-administration (5, 10 and 20 mg/kg) and co-administration with verapamil (*n* = 5).

Parameter	Unit	Intravenous	Mono-Administration			Co-Administration
		2 mg/kg	5 mg/kg	10 mg/kg	20 mg/kg	20 mg/kg
T_max_	h	—	0.5 ± 0.0	0.5 ± 0.0	0.7 ± 0.3	0.7 ± 0.3
C_max_^1^ or C_0_^2^	ng·mL^−1^	11,230.0 ± 149.0	42.3 ± 17.5	63.4 ± 11.4	100.2 ± 42.5	463.6 ± 236.8 **
AUC_0–t_	ng·h·mL^−1^	2401.0 ± 201.9	64.7 ± 44.0	82.3 ± 18.4	148.6 ± 64.8	882.3 ± 490.6 *
AUC_0–∞_	ng·h·mL^−1^	2402.3 ± 201.3	74.7 ± 49.1	84.0 ± 17.0	156.9 ± 65.3	892.0 ± 487.4 *
T_1/2_	h	0.9 ± 0.4	0.8 ± 0.4	0.5 ± 0.1	0.5 ± 0.1	0.7 ± 0.1
MRT_0–∞_	h	0.2 ± 0.0	1.3 ± 0.5	1.0 ± 0.2	1.2 ± 0.1	1.5 ± 0.1
V_z_/F ^1^ or V_z_^2^	L·kg^−1^	0.2 ± 0.0	85.7 ± 21.1	89.0 ± 7.1	112 ± 45.7	31.5 ± 20.3 **
CL/F ^1^ or CL ^2^	L·h^−1^·kg^−1^	0.8 ± 0.1	98.2 ± 77.9	123 ± 27.9	145 ± 54.9	29.2 ± 16.4 **
F	%	—	1.3	0.7	0.6	3.7

^1^ corresponding to oral data; ^2^ corresponding to intravenous data; * *p* < 0.05 was statistically significant; ** *p* < 0.01 was significantly different.

## Data Availability

The data presented in this study are available on request from the corresponding author. The data are not publicly available due to privacy restriction.
